# Survivorship outcomes in patients treated with immune checkpoint inhibitors: a scoping review

**DOI:** 10.1007/s11764-023-01507-w

**Published:** 2024-01-04

**Authors:** Deniz Can Güven, Melissa SY Thong, Volker Arndt

**Affiliations:** 1 Department of Medical Oncology, Hacettepe University Cancer Institute, 06100 Sihhiye, Ankara, Turkey; 2 Health Sciences University, Elazig City Hospital, Elazig, Turkey; 3 Unit of Cancer Survivorship, Division of Clinical Epidemiology and Aging Research, German Cancer Research Center (DKFZ), Heidelberg, Germany

**Keywords:** Immune checkpoint inhibitor, Immunotherapy, Immune-related adverse event, Quality of life, Survivorship

## Abstract

**Background:**

Immune checkpoint inhibitors (ICIs) have become a central part of cancer care. However, the survivorship outcomes in patients treated with ICIs are understudied. Therefore, we conducted a scoping review to evaluate the current status of the field and to establish research gaps regarding survivorship outcomes with ICIs in real-life cohorts.

**Methods:**

We used the Web of Science, PubMed, and Embase databases to systematically filter published studies with real-life cohorts from January 1, 2010, until October 19, 2022. Studies evaluating at least one survivorship outcome in ICI-treated patients were included.

**Results:**

A total of 39 papers were included. Quality of life (QoL) (*n* = 23), toxicity burden (*n* = 16), and psychosocial issues (*n* = 9) were the most frequently evaluated survivorship outcomes. Anti-PD-1/PD-L1 monotherapy and a response to treatment were associated with better QoL. In addition, the ICIs were associated with grade 3 or higher immune-related adverse events (irAEs) in 10–15% and late/long-term irAEs in 20–30% of the survivors. Regarding psychosocial problems, over 30% of survivors showed evidence of anxiety and depression, and 30–40% of survivors reported neurocognitive impairments.

**Conclusion:**

The survivors treated with ICIs have impairments in most survivorship domains. Further research is needed to gather data on the understudied survivorship outcomes like late and long-term effects, fertility, financial toxicity, and return to work in survivors treated with ICIs.

**Implications for Cancer Survivors:**

Available evidence demonstrates that a significant portion of survivors treated with ICIs have a significant toxicity burden, lower QoL than the general population, and a high rate of psychosocial problems.

**Supplementary Information:**

The online version contains supplementary material available at 10.1007/s11764-023-01507-w.

## Introduction

Immune checkpoint inhibitors (ICIs) became one of the fundamental anti-cancer treatments [1] in the last decade due to their unique mechanism of action and improved survival outcomes across several tumors. With ipilimumab being the first ICI to obtain regulatory approval in 2011 for the clinical treatment of metastatic melanoma [2], ICIs have entered treatment algorithms in most advanced tumors. These include renal cell carcinoma [3], non-small cell lung carcinoma [4], gastric and esophageal cancers [5], Hodgkin’s lymphoma [6], small cell lung cancer [7], and urothelial cancer [8].

ICIs have a novel mechanism of releasing the immune system’s brakes that could create long-standing disease control and even cure in a significant portion of patients with advanced cancers, especially in tumors with an immune-active milieu like melanoma and non-small cell lung cancer [9, 10]. Recent studies estimated that almost half of the patients with advanced melanoma treated with ICIs in the first line could be cured of the disease [11]. Phase III clinical trials in non-small cell lung cancer [12] and RCC [13, 14] demonstrated over 30% 5-year survival rates with ICIs. Additionally, ICIs have entered the curative adjuvant or neoadjuvant settings for several tumors [15–17], further expanding the target population of ICIs. These rapid developments, coupled with the long-standing benefits of ICIs in prolonging survival, have created a new group of survivors of advanced cancer treated with ICIs. While improving survival was the predominant treatment goal in advanced cancer, prolonged survival in the last decade highlighted a need for prioritizing survivorship issues like chronic and long-term toxicities in patients with advanced cancer treated with ICIs [18]. Several studies have reported on earlier cessation of ICI in responding patients to prevent toxicities and to maintain a good functional status, considering that a significant portion of the patients could survive a long period or even be cured [19, 20].

Survivorship is a vital but often neglected part of cancer care. According to the National Coalition for Cancer Survivorship (NCCS), all patients with cancer are regarded as survivors from diagnosis to the end of life to as recognition of the potential ongoing problems of persons living with and beyond cancer and to emphasize the necessity to focus on unmet needs throughout the survivorship trajectory [21, 22]. Hence, we will use the term survivors for the rest of the manuscript. With the increasing rate of cancer diagnoses and treatment options, the number of cancer survivors is expected to increase continuously [23]. Recent estimates state that cancer survivors account for about 5% of the general population in Western countries [24].

Despite the significant body of evidence on survivorship outcomes, such as quality of life (QoL) and long-term toxicities, in survivors treated with chemotherapy, radiotherapy, and surgery, the evidence is limited in survivors treated with ICIs. Notwithstanding the remarkable rates of durable tumor response for some survivors, ICI treatments can be toxic. Most reports on immune-related adverse events (irAE) and the effects of irAEs on QoL are from clinical trials, reducing representativeness with real-world cohorts [25]. Additionally, most reports focused on the rates and risk factors of irAEs only instead of evaluating the effects of ICI toxicities on patient-reported outcomes [26, 27].

Recent observational studies involving real-world populations have reported on a range of outcomes related to survivorship, such as QoL, symptom burden, psychological distress, physical activity [28], and financial problems [29], and observed significant disruptions in several domains necessitating dedicated survivorship care in survivors treated with ICIs. However, the available studies varied in sample sizes, study designs, evaluated survivor outcomes, and survivor cohorts. With the increased indications and earlier use of ICIs, an expansion in the number of ICI-treated survivors is expected. Therefore, we systemically reviewed the survivorship outcomes faced by real-world cancer survivors treated with ICIs (as mono- or combination therapy). The scoping review methodology was selected considering the paucity of the quantitative evidence and heterogeneity of the studies. The aim of this scoping review was to evaluate the current status of the field and to create a research agenda by highlighting research gaps.

## Methods

### Literature search

We conducted a scoping review following the Joanna Briggs Institute (JBI) Methods Manual for Evidence Synthesis [30] and reported the results according to PRISMA Extension for Scoping Reviews (PRISMA-ScR) [31]. The study protocol was registered with the Open Science Framework (OSF) at the link https://osf.io/7eg5d. As suggested for scoping reviews, we used the PCC (Population/Concept/Context) instead of PICO: Population, survivors treated with ICIs; Concept, survivorship outcomes; Context, real-life setting [31]. We included studies specifically evaluating the survivorship outcomes in real-life context instead of reports from the clinical trials, in which the main focus was to evaluate the efficacy.

We used the Web of Science, PubMed, and Embase databases to systematically filter the published studies from January 1, 2010, until October 19, 2022. The selected MeSH search terms were as follows: (“health-related quality of life” OR “quality of life” OR “patient reported outcomes” OR “anxiety” OR “depression” OR “psychological” OR “fear of recurrence” OR “financial difficulty” OR “financial problem” OR “return to work” OR “late effect” OR “long-term immune-related adverse event” OR “survivorship” OR “survivor” OR “long-term survivor” OR “financial toxicity” OR “financial burden” OR “symptom burden” OR “psychological distress” OR “psychological well-being” OR “sexual”) AND (“immunotherapy” OR “immune checkpoint inhibitor”). The candidate search terms were selected with the help of a proposed survivorship care framework [32] by the research team. As the survivorship care framework used was very broad [32], we selected to prioritize the survivorship outcomes affecting the daily life of the survivors, and did not include the survivorship outcomes related to decision-making, caregiver burden, and health policy outcomes like coordination of the cancer care.

### Inclusion and exclusion criteria

We included studies that met the following inclusion criteria: (1) prospective or retrospective study to investigate survivorship outcomes in survivors treated with ICI; (2) included a survivor cohort; (3) if survivors treated with targeted therapy and ICI were included, details about the ICI cohort were available; and (4) peer-reviewed full-text available in English. The exclusion criteria of studies were as follows: (1) review articles, case reports, case series, editorials, guidelines, dissertations, and opinion papers; (2) animal and cell-line studies; (3) studies including pediatric patients; (4) immunotherapy other than checkpoint inhibitors; (5) studies reporting on survival outcomes only without the data on the survivorship outcomes; (6) trial protocols; (7) studies evaluating the percentage and risk factors for individual adverse events only without an effect on patient-reported outcomes and late toxicities; (8) qualitative studies; and (9) clinical trials. We used the survivorship definition of the NCCS for the study inclusion and the discussion. In accordance with the NCCS definition, accepting all patients with cancer as cancer survivors after the cancer diagnosis, we included studies independent of the interval between the ICI start time and measurement of the survivorship outcomes.

### Data extraction

Two authors (DCG, MST) extracted the data to an online data sheet following the PRISMA-ScR and JBI guidelines, and any discrepancy between the reviewers was resolved by the senior author (VA). For each study, lead author names, year of publication, study type, sample size, follow-up or evaluation time, tumor type, the ICI agent, evaluated survivorship outcomes, data collection methods, and main results were collected. If the included study evaluated more than one survivorship outcome (for example, both QoL and psychosocial problems), the data for all evaluated survivorship outcomes was extracted, and the individual survivorship outcomes from the included study were discussed in relevant subsections separately. Due to the heterogeneity of the study population and included studies, a narrative synthesis of data was performed. We did not evaluate the individual study qualities in line with the aims of scoping reviews and the recommendations of the JBI manual. Additionally, we searched on the clinicaltrials.gov website to screen ongoing studies evaluating the survivorship outcomes of survivors treated with ICIs.

## Results

### Study selection and descriptive characteristics of the studies

**Fig. 1:**
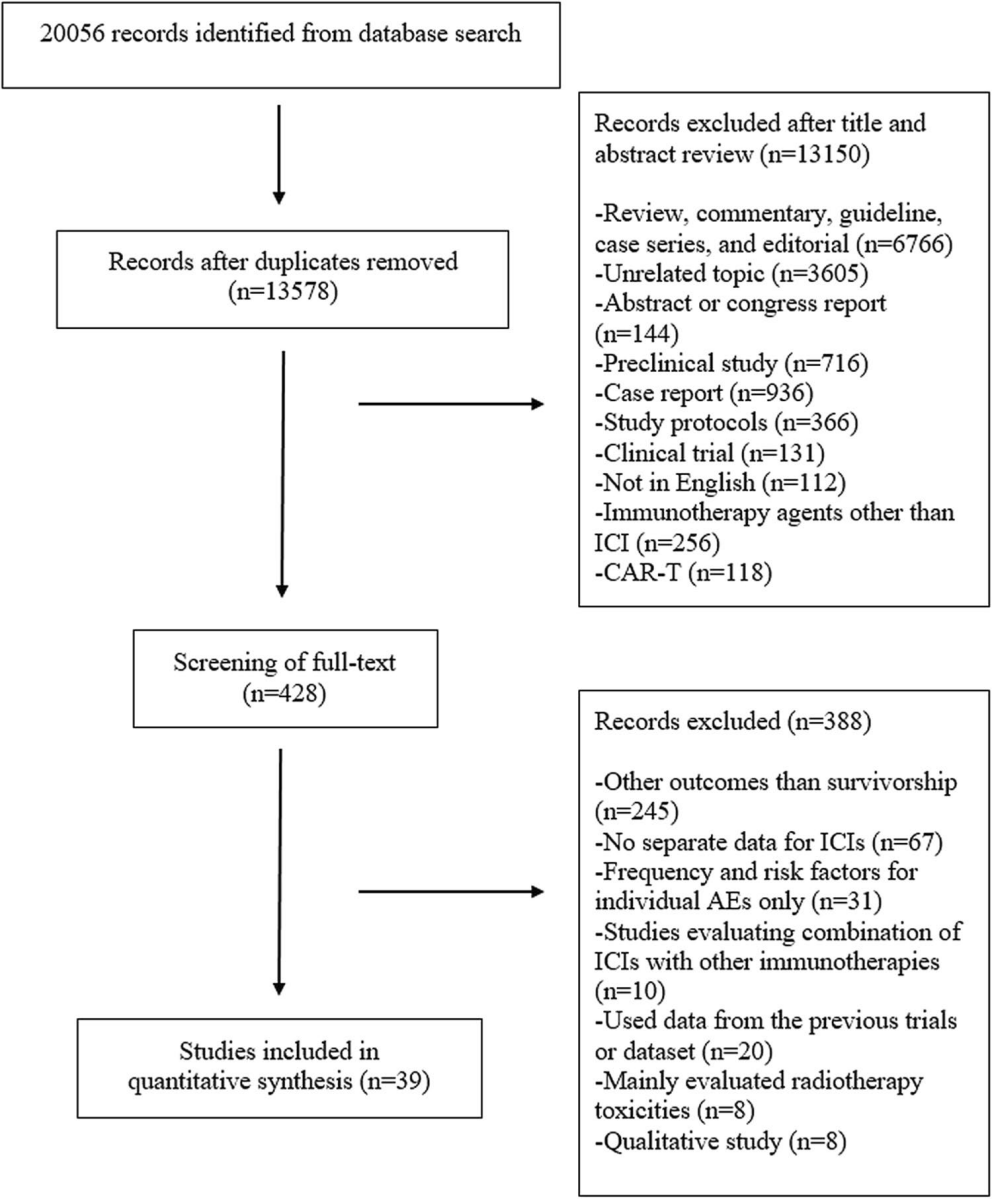
Flowchart for article selection

Our systematic search retrieved 20,056 records. After removing 6478 duplicates, we screened the remaining 13,578 records for inclusion. A total of 13,150 records were excluded after evaluating titles and abstracts. After evaluation of the full texts of the remaining 428 articles, we excluded a further 389 records as these studies evaluated other outcomes like efficacy only (*n* = 245), had no separate data for ICIs in the mixed cohorts (*n* = 67), evaluated combinations of ICIs with other immunotherapy agents (*n* = 10), used data from the databases or previously published datasets (*n* = 20), evaluated frequency and risk factors for individual irAEs only (*n* = 31), evaluated toxicities of radiotherapy in survivors treated with ICIs (*n* = 8), and qualitative studies (*n* = 8). A total of 39 studies from the systematic search were included in the review. The flowchart for article selection is shown in Fig. 1.

Melanoma was the most frequently evaluated tumor (*n* = 14), followed by non-small cell lung cancer (NSCLC) (*n* = 12). ICI monotherapy and ICI-ICI combinations were the most studied treatment regimes, while two studies included survivors treated with ICI plus chemotherapy regimens. Most studies were single center (*n* = 29) and small sample sizes of fewer than 50, while 17 studies had over 100 participants. Prospective cohorts (*n* = 14) and cross-sectional studies (*n* = 14) were the most prevalent study designs. The definition of survivor varied and mainly included predefined time points for ICI duration (6–12–24 months), while two studies only had survivors off therapy. QoL was the most commonly evaluated survivorship issue (*n* = 23), followed by toxicity burden (*n* = 16) and emotional problems (*n* = 9). Most studies were from the USA (*n* = 17), followed by China (*n* = 5) and Germany (*n* = 3). Pembrolizumab (*n* = 22) and nivolumab (*n* = 21) were the most frequently used ICIs in the available studies.

### Assessment of QoL and functional status

A total of 23 studies evaluated QoL using validated questionnaires. The European Organisation for Research and Treatment of Cancer Core Quality of Life questionnaire (EORTC QLQ-C30) was used in 12 studies, followed by the Functional Assessment of Cancer Therapy (FACT) and EuroQol 5 dimensions 5 level (EuroQoL-5D-5L) in four studies each. The sample sizes varied between 18 and 412. Eleven studies were cross-sectional, four were retrospective, and eight were prospective (Table 1). The symptom and toxicity burden was evaluated with the symptom scales of the QoL questionnaires, semi-structured interviews, ECOG and Karnofsky performance status, and CTCAE grading of immune-related adverse events by the treating physician.

**Table 1:** QoL and functional status

Author	Year	Country	Study type	Study design	Sample size (*n*)	Tumor type	ICI type	Inclusion criteria	Outcome	Collected data	Measurement or follow-up time	Study results	Additional comments
Johnson et al. [37]	2015	USA	Single center	Retrospective	33 (24 alive)	Melanoma	Ipi	Survival over 2 years after treatment	Toxicity/functional status	irAEs, physical function (ECOG)	Twenty-four months or later	ECOG 0 or 1 at 23 of 24 survivors/many survivors returned to work/ongoing steroid and thyroid replacement in 33 and 38% of the survivors	One of the earliest reports on this topic/functional independence in almost half of the survivors
Iivanainen et al. [42]	2019	Finland	Multi-center	Retrospective	37	All tumors/all stages	Not specified	Not specified	Outcomes of electronic PRO follow-up	QLQ-C30	Longitudinal follow-up for 6 months, starting with the treatment	The strongest positive relationships among the symptoms identified by correlation analysis were itching and rash, nausea and vomiting, loss of appetite or stomach pain, and cough and shortness of breath	The current study’s findings imply that the data from clinical trials and the real-world symptom information on cancer patients receiving ICI therapy that was gathered using the ePRO program are comparable
Bergerot et al. [43]	2019	USA	Single center	Cross-sectional	60	RCC, prostate, UC	Nivo/Atezo/Nivo+Ipi/Pembro/Avelumab	Not specified	QoL, anxiety and depression, expectation of cure	PROMIS-A, PROMIS-D, FACT-G	At the start of treatment	23% of patients had an inaccurate belief of a very likely cure/accurate expectation of cure was related to a lower prevalence of anxiety (48 vs 82%), while depression rates were similar	
Lai-Kwon et al. [44]	2019	Australia	Single center	Cross-sectional	105 (69 IO)	Melanoma	Pembro/Nivo/Ipi/Ipi+Nivo	Alive and progression-free at least 6 months after the treatment initiation	Survivorship experience	Questionnaire derived from EORTC QLQ-30, EORTC QLQ-MEL38, Social Difficulties Inventory-2, SCNS-SF34/irAEs/Screening for second malignancies	At least 6 months after treatment start	59% ongoing treatment-related toxicity/concerns about long-term side effects (74%), anxiety awaiting results (72%), fear of recurrence (81%), fear of death (64%)/difficulty understanding prognosis (36%)/financial problems in around 20% of the patients/97% felt they should be screened for skin cancer and secondary malignancies	Despite problems, most survivors felt they were able to carry on normally (93%)/high rates of fatigue (90%), insomnia (62%), and joint problems (58%)
Hyatt et al. [45]	2019	Australia	Multi-center	Cross-sectional	55	Melanoma	Not specified	Not specified	Exercise behaviors and fatigue	Exercise behavior questions comprised four items (current level of activity, exercise behaviors (if any), and barriers and facilitators to exercise while receiving immunotherapy) and PROMIS Fatigue–Short Form 7a	N/A	56% of the participants reported exercise during ICI therapy/walking (*n* = 24; 77%) and swimming (*n* = 13; 42%) were the most common activities among participants who reported exercising during treatment/fatigue was most commonly self-reported side effects of ICI (*n* = 34, 62%), followed by dermatological side effects (*n* = 33, 60%), such as itch or rash	More than half of patients (64%) reported that immunotherapy affected their ability to exercise
McLouth et al. [46]	2020	USA	Multi-center	Cross-sectional	60	NSCLC	Pemb, Nivo, Atezo	Not specified	Toxicity, irAE, HRQoL, performance status, financial toxicity	PRO-CTCAE, EORTC QLQ-C30, ECOG	Median 20.8 weeks after treatment start	Highest EORTC symptoms for fatigue, insomnia, dyspnea, and financial concerns/worse global health, pain, and CTCAETM for patients with ECOG 2/3	
Rogiers et al. [35]	2020	Belgium	Single center	Prospective	18	Melanoma	Ipi	Alive after 2 years of ICI	NCF, psychosocial outcome, HRQoL, irAE	HADS, EORTC QLQ-C30, FSS, CFQ, Interview, Neurocognitive Function Tests	Twenty-four months or later	Even after cessation of ICI, 41% of survivors have impaired NCF, high levels of anxiety and depression, and decreased QoL	One case of autoimmune hypophysitis manifests itself as mood disorder and suicidal thoughts
Joseph et al. [33]	2020	USA	Multi-center	Prospective	412	Melanoma	Pemb/Ipi+Nivo	Not specified	QoL	EORTC QLQ-C30, EQ-5D-5L	Longitudinal follow-up for 6 months, starting with the treatment	QoL was increased with pembro at the 24th week, while remained stable with Nivo+Ipi/Both regimens improved QoL in responding patients/QoL decreased with Nivo+Ipi and remained stable with Pembro in non-responding patients	ICI monotherapy was related to better QoL compared to a combination
Rogiers et al. [35]	2020	Belgium	Single center	Prospective	25	Melanoma	Pemb	Alive and progression-free at least 6 months after the treatment initiation	QoL, NCF, emotional burden	EORTC QLQ-C30, HADS, FSS, Cogstate test battery, Semi-structured interview	Longitudinal follow-up for 1 year (after at least 6 months of survival with ICIs)	Lower mean EORTC QLQ-C30 scores than the general population/32% of survivors had cognitive impairment in any of two evaluation time points/higher levels of fatigue, pain, and insomnia problems/cancer-related PTS in 48% and severe anxiety 32%	35 of 141 patients were long-term survivors/48% had unresolved irAEs
Patrinely et al. [39]	2020	USA	Single center	Retrospective	217	MM, RCC, NSCLC	Pemb/Nivo/Atezo/Ave/Ipi+Nivo	Survival over 2 years after treatment	Toxicity/HRQoL/body composition/functional status	FACT-G/IESR/NCCN-DT/Body Composition (CT)/irAEs	Twenty-four months or later	No dif. in BP, glucose, and BMI/increased adiposity and muscle mass/20% chronic IRAEs/younger age and need for subsequent therapy > worse QoL	Used body composition data from baseline and follow-up/acceptable QoL but increased risk in younger survivors/significant chronic irAE burden
Mamoor et al. [26]	2020	USA	Single center	Cross-sectional	90	Melanoma	Ipi+Nivo/Pembro/Ipi/Nivo/Durva	Alive at 12 months after the treatment initiation	Symptom burden/QoL	EORTC QLQ-C30, EuroQoL, PRO-CTCAE, FSS	At least 12 months or later after the start of therapy	Fatigue in 28%, sleeping difficulties and muscle-joint complaints in around 10–15%, and anxiety-depression in 40% of the survivors	Acceptable QoL for most survivors/mobility problems seemed to increase with increased survival after treatment
O'Reilly et al. [47]	2020	UK	Single center	Cross-sectional	84	Melanoma	Pembro/Nivo/Ipi/Ipi+Nivo/Clinical Trial	Alive and progression-free at 12 months after the treatment initiation	Toxicity, QoL, exposure to immunosuppressive agents	SF-36, irAEs	Twelve months or later	Fourteen survivors required beyond steroids for irAEs, and 12 patients developed irAEs after discontinuation/lower QoL in melanoma survivors in physical and social functioning and general health domains	A trend towards inferior QoL scores in patients previously exposed to ipilimumab
Thom et al. [29]	2021	USA	Single center	Cross-sectional	106	Melanoma	Ipi+Nivo/Ipi/Pembro&Nivo/Durva	Alive at 12 months after the treatment initiation	Financial toxicity, QoL	COST, EORTC QLQ-30	The median time since ICI started was 36.4 months	Financial difficulties in 23% of the patients/higher financial toxicity (*p* < 0.001) and financial difficulties (*p* = 0.01) in survivors <65 years of age/financial toxicity had a significant correlation with global QoL and social, emotional, and role subscales	The first study providing quantitative data on the association between financial toxicity and QoL in an immunotherapy cohort
Jim et al. [34]	2021	USA	Multi-center	Prospective	226	NSCLC	Pemb/Nivo/Atezo/Durva	Not specified	QoL, toxicity	EORTC QLQ-C30, FACT-G, irAE	N/A (47% were in the 2–6 months of treatment)	No differences were observed by ICI regimen in overall quality of life or subscales. Worse overall QoL, emotional well-being, and functional well-being than the US population	The three most common symptomatic toxicities were fatigue (85%), aching joints (63%), and aching muscles (57%)
Zhang et al. [41]	2022	China	Multi-center	RCT	278	GEC, lung, PDAC, CRC, breast, CNS, HCC, RCC, other	Not specified	Not specified	irAE, QoL, treatment discontinuation, ED visits	CTCAE, EORTC QLQ-C30	Longitudinal follow-up for 6 months, starting with the treatment	ePROapp group demonstrated lower severe irAEs, fewer ED visits, lower rates of treatment discontinuation, and a higher QoL	
Yin et al. [48]	2022	China	Single center	Prospective	124	NSCLC	Camrelizumab, sintilimab, other	Not specified	QoL, CWS, toxicity	Karnofsky, CWS, EORTC QLQ, irAE	After 4–6 courses of treatment or upon cancer progression	60% of the survivors had high FCR at baseline/high FCR was associated with lower QoL	High levels of FCR were related to lower response rates
Schulz et al. [27]	2022	Germany	Multi-center	Cross-Sectional	200	Melanoma, SCC, MCC	PembNivo, Ipi+Nivo, Ipi, avelumab, cemiplimab	Cessation of ICIs at least 12 weeks before treatment	iRAE, HRQoL	CTCAE, EuroQol 5D-5L	At least 12 weeks after ICI cessation	Long-term/chronic irAEs in 51.9%/35.5% of the outpatient ICI survivors/HRQoL in survivors with long-term or chronic irAEs were lower than patients without long-term or chronic irAEs and were similar to patients with chronic autoimmune disorders	
Li et al. [49]	2022	China	Single center	Prospective	67	NSCLC	Not specified (CT+IO)	Not specified	QoL, depression	EORTC QLQ-C30, Zung SDS	N/A	Patients with depression had significantly lower ORR, DCR, and PFS compared to survivors without depression/QoL was higher in survivors without depression	
Bi et al. [50]	2022	China	Single center	Cross-sectional	108	NSCLC	Camrelizumab, sintilimab, Pemb, Atezo, Durva	Not specified	QoL, toxicity	EORTC QLQ-C30 and NCCN-DT	Before treatment and at the progression	Lower ORR, DCR, and shorter PFS in survivors with psychological distress than those with no psychological distress	
Koldenhof et al. [51]	2022	Netherlands	Single center	Retrospective	162	Melanoma, NSCLC	Pemb/Nivo/Atezo/Durva	Not specified	PRO	HRQoL, USD-I scores	Longitudinal follow-up for 12 months, starting with the treatment	The most common patient-reported symptoms were inactivity, fatigue, pain, cough, and sleeping problems/symptom prevalence decreased during treatment/survivors generally reported a low influence on QoL with treatment	Used data from an ICI-tailored PROM in routine clinical practice
Tolstrup et al. [40]	2022	Denmark	Single center	RCT	138	Melanoma	Ipi, Pemb, nivolumab, Ipi b/nivolumab	Not specified	Patient-reported outcome, toxicity, irAE, HRQoL, performance status	EuroQol EQ-5D Index, FACT-M, irAE, ECOG	Longitudinal follow-up for 12 months	Using ePROs improved HRQoL measured by EQ-5D-5L at follow-up/similar changes in functional status irrespective of ePRO use	Evaluated the impact of using ePROs on HRQoL in melanoma survivors treated with ICIs
Wong et al. [52]	2022	USA	Multi-center	Cross-sectional	93	NSCLC	Not specified	Not specified	Toxicity, performance, depression, anxiety, life space mobility	LSA, ECOG, MHI-13, MoCA	Before treatment, and 1, 2, 4, and 6 months after the start of treatment	Patients who have high anxiety during the pretreatment period are more likely to have lesser LSA scores; age and performance status are not associated with functional decline	LAS changes did not differ between patients who got immunotherapy and patients who got chemotherapy; pretreatment fatigue is not associated with a higher LAS score decrease
Singhal et al. [38]	2022	USA	Multi-center	Cross-sectional	87	NSCLC	Not specified	Not specified	Performance status, daily instrumental activities, HRQoL	Karnofsky PS, IADL, EORTC QLQ-C30, LSA	Before treatment, and 2, 4, and 6 months after the start of treatment	Older survivors have declined functional scores, and only a small percentage of these survivors can be recovered in 6 months. Clinicians should use at least two different tools to detect early deterioration in functionality	LSA may be an early tool to detect functional decline in this patient group instead of IADL and KPS. Also, interestingly, in this study, KPS and LSA are not statistically associated with each other

Abbreviations: *Atezo*, atezolizumab; *BMI, body mass index*, *BP*, blood pressure; CRC, colorectal cancer; CTCAE, *Common Terminology Criteria for Adverse Events*; *CWS*, Cancer Worry Scale; *DCR*, disease control rate; *ECG*, electrocardiogram; *ECOG*, Eastern Cooperative Oncology Group; *ED*, e*mergency department*; *EORTC QLQ-C30 PF*, European Organisation for Research and Treatment of Cancer Quality of Life Questionnaire Physical Functioning; *FACT-M*, Functional Assessment of Cancer Therapy Melanoma; *FCR*, fear of *cancer* recurrence; *FSS*, Fatigue Severity Scale; *HADS*, Hospitalization Anxiety and Depression Scale; *HCC, hepatocellular carcinoma; HRQoL*, *health-related quality of life; IADL*, instrumental activities of daily living; *ICI*, immune checkpoint inhibitor; *Ipi*, ipilimumab; *irAE*, immune-related adverse events; *KPS*, Karnofsky Performance Scale; *LSA*, Life-Space Assessment; *MoCA*, *Montreal Cognitive Assessment*; *NCCN-DT, NCCN National Comprehensive Cancer Network Distress Thermometer*; *NCF*, neurocognitive function; *Nivo*, nivolumab; *NSCLC*, non-small cell *lung cancer; ORR*, overall response rate; *PDAC*, pancreatic ductal adenocarcinoma; *PEMB, pembrolizumab; PFS,* progression-free survival; *PRO, patient*-reported outcome; *PROMIS*, Patient-Reported Objective Measurement Information System; *RCC*, renal cell cancer; *RCT*, *randomized controlled trial*

Most studies reported effects on the multiple QoL domains, and fatigue was the most frequently affected QoL domain, with fatigue rates of up to 90% reported with ICIs. However, a prospective study reported improved QoL with pembrolizumab monotherapy in melanoma survivors [33]. Furthermore, the rate of fatigue was very variable across the studies. While Mamoor et al. reported a 28% fatigue rate in melanoma survivors who were alive at least 12 months after treatment [26], the fatigue rate was 85% in a study including survivors with NSCLC without a time-prespecified treatment frame [34]. Two studies compared the QoL of the survivors treated with ICIs to the general population [34–36], and significantly worse QoL and functional well-being compared to the general population were noted in both studies. However, over 90% of the survivors with long-term disease control under ICIs were able to carry on routine daily activities. Similar to QoL, the outcomes regarding functional decline varied according to the evaluation time. Johnson et al. reported an ECOG status of 0 or 1 in 23 of the 24 patients with long-term survival with ICIs. The cohort’s median age was 60 years and most survivors returned to work [37]. In contrast, Singhal et al. reported a functional decline in 70% of the older adults (≥65 years) with lung cancer and recovery in only 13% of the survivors [38].

#### Factors associated with QoL

The factors affecting the QoL were evaluated in several studies. Jim et al. observed a similar QoL with different ICI regimens (all monotherapy) [34], while anti-PD-1 monotherapy was associated with better QoL compared to anti-PD-1/anti-CTLA-4 combination in survivors with melanoma in the study by Joseph et al. [33]. It should be noted that QoL was improved in survivors who responded to ICI therapy (mono- or in combination), while in survivors without a response to ICIs, QoL remained stable [33]. In addition to the ICI regimen, younger age and the need for subsequent therapy were associated with lower QoL in one study [39]. Schulz et al. observed that the QoL was lower in survivors who developed chronic and long-term irAEs than in survivors without these adverse events and similar QoL in patients with chronic autoimmune disorders and survivors with long-term and chronic irAEs [27]. In addition to these studies evaluating the denominators of lower QoL, two studies reported improvements in the QoL in survivors followed up with an electronic patient-reported outcome (ePRO) tool when compared with traditional care follow-up [40, 41].

### Toxicity burden

The toxicity burden was most commonly evaluated as irAEs. Grade 3 or higher irAEs were observed in around 10–15% of the survivors, while in most studies, over 50% of the survivors had any grade irAEs. The prevalence of late irAEs was reported in three studies (Table 2). The definition of late and persistent irAEs in these studies differed from the recent Society for Immunotherapy of Cancer (STIC) recommendations for irAE terminology [53]. Nigro et al. defined late irAEs as irAEs occurred after >12 months of treatment and reported late irAEs in 30.1% of the survivors treated with at least 12 months of anti-PD-1/PD-L1 inhibitors [54]. Hall et al. reported irAEs in 24% of the survivors after at least 6 months of treatment and defined these irAEs as late irAEs [55]. Hsu et al. defined irAEs lasting over 12 months as long-term irAEs. They reported long-term irAEs in 23.7% of the survivors with non-small cell lung cancer treated with ICIs and late-onset irAEs (occurred at least 1 year after treatment initiation) in 16% of the patients [56]. In contrast, Argnani et al. evaluated 32 survivors with non-Hodgkin lymphoma and reported no late irAEs [57].

**Table 2:** Toxicity burden

Author	Year	Country	Study type	Study design	Sample size (*n*)	Tumor type	ICI type	Inclusion criteria	Outcome	Collected data	Measurement or follow-up data	Study results	Additional comments
Johnson et al. [37]	2015	USA	Single center	Retrospective	33 (24 alive)	Melanoma	Ipi	Survival over 2 years after treatment	Toxicity/functional status	irAEs, physical function (ECOG)	Twenty-four months or later	ECOG 0 or 1 at 23 of 24 survivors/many patients returned to work/ongoing steroid and thyroid replacement in 33 and 38% of the survivors	One of the earliest reports on this topic/functional independence in almost half of the survivors
Hall et al. [55]	2020	USA	Single center	Retrospective	159	Melanoma, NSCLC, RCC, HNSCC, TCC	Pemb/Nivo/Atezo	Use of ICIs for at least 6 months	Toxicity	Late irAE	Six months or later	Thirty-eight survivors experienced late irAEs (24%) after 6 months of treatment, while 11 of survivors experienced two or more late irAEs. New or worsening of hypothyroidism was the most common clinically significant late irAE	No association was observed between disease response and the development of late irAEs
Nigro et al. [54]	2020	Italy	Multi-center	Retrospective	436	NSCLC, melanoma, RCC, and others	Pemb/Nivo/Atezo	Patients treated with at least 12 months of ICIs	Late irAEs	CTCAE	Twelve months or later	223 survivors experienced any grade early-irAEs (51.1%), while 132 experienced any grade late irAEs (30.3%)/in 78% of the patients, late irAEs were different denovo irAEs	No statistically significant difference in PFS or OS according to the presence of early or late irAEs
Patrinely et al. [39]	2020	USA	Single center	Retrospective	217	MM, RCC, NSCLC	Pemb/Nivo/Atezo/Ave/Ipi+Nivo	Survival over 2 years after treatment	Toxicity/HRQoL/body composition/functional status	FACT-G/IESR/NCCN-DT/body composition (CT)/irAEs	Twenty-four months or later	No dif. in BP, glucose, and BMI/increased adiposity and muscle mass/20% chronic irAEs/younger age and need for subsequent therapy > worse QoL	Used body composition data from baseline and follow-up/acceptable QoL but increased risk in younger survivors/significant chronic irAE burden
O'Reilly et al. [47]	2020	UK	Single center	Cross-sectional	84	Melanoma	Pembro/Nivo/Ipi/Ipi+Nivo/Clinical Trial	Alive and progression-free at 12 months after the treatment initiation	Toxicity, QoL, exposure to immunosuppressive agents	SF-36, irAEs	Twelve months or later	Fourteen survivors required beyond steroids for irAEs, and 12 patients developed irAEs after discontinuation/Lower QoL in melanoma survivors in physical and social functioning and general health domains	A trend towards inferior QoL scores in patients previously exposed to ipilimumab
McLouth et al. [46]	2020	USA	Multi-center	Cross-sectional	60	NSCLC	Pemb, Nivo, Atezo	Not specified	Toxicity, irAE, HRQoL, performance status, financial toxicity	PRO-CTCAE, EORTC QLQ-C30, ECOG	Median 20.8 weeks after treatment start	Highest EORTC symptoms for fatigue, insomnia, dyspnea, and financial concerns/worse global health, pain, and CTCAETM for survivors with ECOG 2/3	
Salzmann et al. [58]	2021	Germany	Single center	Cross-sectional	25	Melanoma (cutaneous, uveal), cSCC	Various (Nivo, Ipi, Pembro, Nivo+Ipi, PD-1+/-LAG-3, PD-1+/-CTLA-4)	Initiation of ICIs at least 3 months before inclusion	Male fertility, toxicity	Spermiogram	Three months or later	Normal spermiogram in 18/22 survivors and normal sexual function and activity in all participants	The cause of azoospermia could be ICI-related in two survivors (2/22)
Jim et al. [34]	2021	USA	Multi-center	Prospective	226	NSCLC	Pemb/Nivo/Atezo/Durva	Not specified	QoL, toxicity	EORTC QLQ-C30, FACT-G, irAE	N/A (47% were in the 2–6 months of treatment)	No differences were observed by ICI regimen in overall quality of life or subscales. Worse overall QoL, emotional well-being, and functional well-being than the US population	The three most common symptomatic toxicities were fatigue (85%), aching joints (63%), and aching muscles (57%)
Hsu et al. [56]	2022	USA	Single center	Retrospective	114	NSCLC	Nivo/Pembro/Durva/MDX-1105/IO combinations	Alive at 12 months after the treatment initiation	Toxicity	irAE	Twelve months or later	Chronic irAEs, late-onset, and long-term irAEs in 37.7%, 16%, and 23.7% of the survivors, respectively	High rates of steroid (45%) and additional immunosuppressant use (16%) in 31 patients with unresolved irAEs/toxicity in the first year correlated with long-term toxicity (*ρ* = 0.72, *p* < 0.001)
Tong et al. [59]	2022	Canada	Single center	Retrospective	161	Melanoma (adjuvant or metastatic)	Pembro, Nivo, Ipi, Nivo+Ipi, other	Not specified	irAE	CTCAE, irAE	Median follow-up: 22.6 months	Permanent irAEs in 65.6% and 34.9% of the survivors treated with dual monotherapy and ICI monotherapy, respectively/12.4% grades 3–4 and 2.5% grade 5 irAEs	Most of the permanent irAEs were related to endocrine or skin-related AEs
Zhang et al. [41]	2022	China	Multi-center	RCT	278	GEC, lung, PDAC, CRC, breast, CNS, HCC, RCC, other	Not specified	Not specified	irAE, QoL, treatment discontinuation, ED visits	CTCAE, EORTC QLQ-C30	Longitudinal follow-up for 6 months, starting with the treatment	ePROapp group demonstrated lower severe irAEs, fewer ED visits, lower rates of treatment discontinuation, and a higher QoL	
Yin et al. [48]	2022	China	Single center	Prospective	124	NSCLC	Camrelizumab, sintilimab, other	Not specified	QoL, CWS, toxicity	Karnofsky, CWS, EORTC QLQ, irAE	After 4–6 courses of treatment or upon cancer progression	60% of the survivors had high FCR at baseline/high FCR was associated with lower QoL	High levels of FCR were related to lower response rates
Schulz et al. [27]	2022	Germany	Multi-center	Cross-sectional	200	Melanoma, SCC, MCC	Pemb, Nivo, Ipi+Nivo, Ipi, avelumab, cemiplimab	Cessation of ICIs at least 12 weeks before treatment	iRAE, HRQoL	CTCAE, EuroQol 5D-5L	At least 12 weeks after ICI cessation	Long-term/chronic irAEs in 51.9%/35.5% of the outpatient ICI survivors/HRQoL in patients with long-term or chronic irAEs were lower than survivors without long-term or chronic irAEs and were similar to patients with chronic autoimmune disorders	
Bi et al. [50]	2022	China	Single center	Cross-sectional	108	NSCLC	Camrelizumab, sintilimab, Pemb, Atezo, durvalumab	Not specified	QoL, toxicity	EORTC QLQ-C30 and NCCN-DT	Before treatment and at the progression	Lower ORR, DCR, and shorter PFS in survivors with psychological distress than those with no psychological distress	
Tolstrup et al. [40]	2022	Denmark	Single center	RCT	138	Melanoma	Ipi, Pemb, Nivo, Ipi /Nivo	Not specified	Patient-reported outcome, toxicity, irAE, HRQoL, performance status	EuroQol EQ-5D Index, FACT-M, irAE, ECOG	Longitudinal follow-up for 12 months	Using ePROs improved HRQoL measured by EQ-5D-5L at follow-up/similar changes in functional status irrespective of ePRO use	Evaluated the impact of using ePROs on HRQoL in melanoma survivors treated with ICIs
Wong et al. [52]	2022	USA	Multi-center	Cross-sectional	93	NSCLC	Not specified	Not specified	Toxicity, performance, depression, anxiety, life space mobility	LSA, ECOG, MHI-13, MoCA	Before treatment and 1, 2, 4, and 6 months after the start of treatment	Survivors who have high anxiety at the pretreatment period are more likely to have lesser LSA scores, and age and performance status are not associated with functional decline	LAS changes did not differ between patients who got immunotherapy and survivors who got chemotherapy; pretreatment fatigue is not associated with a higher LAS score decrease

Abbreviations: *AKI*, acute kidney injury; *Atezo*, atezolizumab; *BNP, brain* natriuretic peptide; *BMI, body mass index*, *BP*, blood pressure; CCI, *Charlson* Comorbidity Index; *CCP*, cyclic citrullinated peptide; *CK*, creatine kinase; *CKD*, chronic kidney disease; *CRC*, colorectal cancer; *CTCAE*, *Common Terminology Criteria for Adverse Events*; *CWS*, Cancer Worry Scale; *DCR*, disease control rate; *ECG*, electrocardiogram; *ECOG*, Eastern Cooperative Oncology Group; *EORTC QLQ-C30 PF*, European Organisation for Research and Treatment of Cancer Quality of Life Questionnaire Physical Functioning; *FABP*, *fatty-acid-binding protein; FACT-M*, Functional Assessment of Cancer Therapy Melanoma; *FCR*, fear of *cancer* recurrence; *GFR*, glomerular filtration rate; *GVHD*, *graft-versus-host disease; HCC, hepatocellular carcinoma; HNSCC, head and neck squamous cell carcinoma*; *HRQoL*, *health-related quality of life; ICI*, immune checkpoint inhibitor; *Ipi*, ipilimumab; *irAE*, immune-related adverse events; *KPS*, Karnofsky Performance Scale; *LSA*, Life-Space Assessment; *LVEF*, *left ventricular ejection fraction*; *MoCA*, *Montreal Cognitive Assessment*; *NCCN-DT, National Comprehensive Cancer Network Distress Thermometer*; *NCF*, neurocognitive function; *Nivo*, nivolumab; *NSCLC*, non-small cell *lung cancer; ORR*, overall response rate;*OS*, overall survival; *PDAC*, pancreatic ductal adenocarcinoma; *PEMB, pembrolizumab; PFS,* progression-free survival; *PRO, patient*-reported outcome; *RCC*, renal cell cancer; *RCT*, *randomized controlled trial; SJS, Stevens-Johnson syndrome*; *TEN*, toxic epidermal necrolysis; *TFT, thyroid* function tests; *TRAE, treatment-related adverse event; TTE, transthoracic* echocardiogram; *VTE*, *venous thromboembolism*

### Psychological and neurocognitive problems

Eleven studies specifically evaluated psychological outcomes such as anxiety and depression with dedicated questionnaires (Table 3). All studies reported a high rate of anxiety and depression, ranging from 30 to 82%. While four studies cross-sectionally assessed anxiety and depression rates, two studies conducted longitudinal evaluations, and both reported decreasing levels of anxiety and depression during ICI treatment. The Hospital Anxiety and Depression Scale (HADS) was the most frequently used measurement (*n* = 5), followed by structured interviews and the distress thermometer. Sample sizes were mainly between 100 and 200, and almost all studies evaluated survivors with non-small cell lung cancer or melanoma. In addition to the prevalence of anxiety, depression, and psychological distress, two studies reported significantly lower overall response rates to ICIs in survivors with depression or psychological distress [49, 50]. Lastly, Thewes et al. reported fear of recurrence in 60% of the survivors with non-small cell lung cancer treated with ICIs, and another study with a limited sample size (73 survivors, of whom only nine were treated with ICIs) reported significantly higher fear of recurrence in adolescents and young adults treated with ICIs compared to survivors treated with other treatments [60].

**Table 3:** Psychological and neurocognitive problems

Author	Year	Country	Study type	Study design	Sample size (*n*)	Tumor type	ICI type	Inclusion criteria	Outcome	Collected data	Measurement or follow-up time	Study results	Additional comments
Kovács et al. [62]	2014	Hungary	Single center	Prospective	28 (10 IO)	Melanoma	Ipilimumab	Not specified	Depression, anxiety	Zung Self-Rating Depression Scale, STAI	Every 3 weeks during ipilimumab treatment/at baseline, 1st, 3rd, and 6th month of interferon therapy	The presence of depression was not increased with ipilimumab in contrast to interferon/anxiety levels transiently increased with ipilimumab	
Thewes et al. [60]	2018	Netherlands	Single center	Cross-sectional	73 (9 ICI)	All tumors/all stages	Not specified	AYA patients consulted to a dedicated AYA team	Fear of Cancer Recurrence, Anxiety, Depression, Distress, HRQoL	CWS, HADS, HRQoL	N/A	Higher fear of recurrence in AYA with cancer treated with ICIs	No association has been found between FCR and physical HRQoL, while anxiety, depression, and functioning were worse in patients with FCR
Bergerot et al. [43]	2019	USA	Single center	Cross-sectional	60	RCC, prostate, UC	Nivo/Atezo/Nivo+Ipi/Pembro/avelumab	Not specified	QoL, anxiety and depression, expectation of cure	PROMIS-A, PROMIS-D, FACT-G	At the start of treatment	23% of survivors had an inaccurate belief of a very likely cure/accurate expectation of cure was related to a lower prevalence of anxiety (48 vs 82%), while depression rates were similar	
McFarland [63]	2019	USA	Single center	Cross-sectional	109 (35 ICI)	SCLC/NSCLC	Not specified	Not specified	Psychological outcomes	Distress Thermometer and Problem List, HADS	At least 1 month of treatment	Targeted treatments and immunotherapy are linked to lower levels of depression and inflammation in lung cancer survivors, but there were no differences in distress or anxiety levels	Compared to immunotherapy or targeted medicines, chemotherapy is more closely linked to depression and increased inflammation
Rogiers et al. [36]	2020	Belgium	Single center	Prospective	25	Melanoma	Pembrolizumab	Alive and progression-free at least 6 months after the treatment initiation	QoL, NCF, emotional burden	EORTC QLQ-C30, HADS, FSS, Cogstate test battery, semi-structured interview	Longitudinal follow-up for 1 year (after at least 6 months of survival with ICIs)	Lower mean EORTC QLQ-C30 scores than the general population/32% of survivors had cognitive impairment in any of two evaluation time points/higher levels of fatigue, pain, and insomnia problems/cancer-related PTS in 48% and severe anxiety 32%	35 of 141 patients were long-term survivors/48% had unresolved irAEs
Rogiers et al. [35]	2020	Belgium	Single center	Prospective	18	Melanoma	Ipilimumab	Alive after 2 years of ICI	NCF, psychosocial outcome, HRQoL, irAE	HADS, EORTC QLQ-C30, FSS, CFQ, Interview, Neurocognitive Function Tests	Twenty-four months or later	Even after cessation of ICI, 41% of survivors have impaired NCF, high levels of anxiety and depression and decreased QoL.	One case of autoimmune hypophysitis manifests itself as mood disorder and suicidal thoughts
Wiens et al. [64]	2021	Germany	Single center	Prospective	113	Melanoma (adjuvant or metastatic)	Ipilimumab, nivolumab, pembrolizumab, Ipi+Nivo-	Not specified	Stress, psychooncologic support need, toxicity	DT, HSI, Self-Assessment Questionnaire	Before initiation of ICI, after 6–8 weeks, and after 12–14 weeks	Higher rates of distress at the start of ICI treatment and decreased stress level throughout therapy/increased distress in female patients and patients who had AEs	Higher rates of toxicities with dual ICIs/site of the disease is not associated with stress levels
Bodd et al. [65]	2022	USA	Single center	Retrospective	152	NSCLC	Not specified	Not specified	Patient-reported distress	NCCN Distress Thermometer (DT) and its 39-item Problem List	Longitudinal monthly follow-up	The most frequent sources of distress were fatigue, pain, and breathing, which remained high longitudinally	
Invitto et al. [61]	2022	Italy	Single center	Cross-sectional	154 (43 IO)	All Tumors	Not specified	Geriatric patients	Olfactory perception	The Sniffin’ Sticks Screening 12	N/A	A significant decrease in olfactory perception ability following ICIs	All patients were symptomatic at the time of diagnosis of hypophysitis. The most common presentations were fatigue (86%) and headache (43%)
Li et al. [49]	2022	China	Single center	Prospective	67	NSCLC	Not specified (CT+IO)	Not specified	QoL, depression	EORTC QLQ-C30, Zung SDS	N/A	Survivors with depression had significantly lower ORR, DCR, and PFS compared to patients without depression/QoL was higher in patients without depression	
Andersen et al. [66]	2022	USA	Single center	Prospective	82	NSCLC	Not specified	Survival over 2 years after treatment	Depression, anxiety	Patient Health Questionnaire-9, Generalized Anxiety Disorder-7	Longitudinal follow-up for 24 months	Decreased depression and anxiety symptoms with time since diagnosis/the 2-year trajectory of depressive symptoms was significantly associated with cancer survival (HR = 1.09, 95% CI = 1.03–1.15, *p* = .002)	First study to test the interaction of a longitudinal trajectory of psychological symptoms and cancer survival
Wong et al. [52]	2022	USA	Multi-center	Cross-sectional	93	NSCLC	Not specified	Not specified	Toxicity, performance, depression, anxiety, life space mobility	LSA, ECOG, MHI-13, MoCA	Before treatment, and 1, 2, 4, and 6 months after the start of treatment	Survivors who have high anxiety during the pretreatment period are more likely to have lesser LSA scores, and age and performance status are not associated with functional decline	LAS changes did not differ between patients who got immunotherapy and patients who got chemotherapy; pretreatment fatigue is not associated with a higher LAS score decrease
Xie et al. [67]	2022	China	Single center	Prospective	126	Not specified	Not specified	Not specified	Anxiety, depression	HADS	Before treatment and after 1, 2, and 3 courses of treatment	Increased rates of anxiety and depression during treatment/A 54% anxiety and depression rate after three courses of treatment	
Khalil et al. [68]	2022	Lebanon	Single center	Prospective	44 (32 and 24 for first and second analysis)	All tumors	Pemb/Nivo/Atezo	Not specified	Neuropsychiatric adverse events (NPAEs)	Brief Psychiatric Rating Scale (BPRS), Herth Hope Index (HHI)	Before and after 1 and 3 months of treatment	No changes in BPRS total score/decreases in motor retardation and nervousness subscores of BPRS	
Valentine et al. [28]	2022	USA	Single center	Prospective	186	NSCLC	Not specified	Not specified	Disease perception profile, symptom severity, psychological status	Patient interviews, Physical symptom severity, Cough Severity, Anxiety Depression Severity tests	Before treatment	Negative illness perception is associated with a worse disease burden and this group is in need of prompt referral to psychological support	

Neurocognitive problems were addressed in four studies. Rogiers et al. evaluated neurocognitive function (NCF) in long-term survivors previously treated with pembrolizumab and ipilimumab and had their treatment ceased after long-term disease control [35]. The authors reported NCF disturbances, such as attention, memory, and executive function, in 32% and 41% of the survivors treated with pembrolizumab and ipilimumab, respectively. In addition to these problems, Invitto et al. reported an increasing rate of olfactory impairments and anosmia in elderly survivors treated with ICIs compared to survivors treated with CT or geriatric control subjects [61].

Abbreviations, *Atezo*, atezolizumab; *BPRS*, Brief Psychiatric Rating Scale; *CTCAE*, *Common Terminology Criteria for Adverse Events*; *DCR*, disease control rate; *ECG*, electrocardiogram; *ECOG*, Eastern Cooperative Oncology Group; *EORTC QLQ-C30 PF*, European Organisation for Research and Treatment of Cancer Quality of Life Questionnaire Physical Functioning; *FACT-M*, Functional Assessment of Cancer Therapy Melanoma; *HADS*, Hospitalization Anxiety and Depression Scale; *HRQoL*, *health-related quality of life; ICI*, immune checkpoint inhibitor; *Ipi*, ipilimumab; *irAE*, immune-related adverse events; *LSA*, Life-Space Assessment; *MoCA*, *Montreal Cognitive Assessment*; *NCCN-DT, National Comprehensive Cancer Network Distress Thermometer*; *NCF*, neurocognitive function; *Nivo*, nivolumab; *NPAE*, neuropsychiatric adverse event; *NSCLC*, non-small cell *lung cancer; ORR*, overall response rate; *OS*, overall survival; *Pemb, pembrolizumab; PFS,* progression-free survival; *RCC*, renal cell cancer; *RCT*, *randomized controlled trial; STAI*, State-Trait Anxiety Inventory

### Financial toxicity

Financial toxicity was evaluated with a dedicated questionnaire (The Comprehensive Score for Financial Toxicity (COST)) in two studies. Thom et al. demonstrated that 23% of the survivors reported financial difficulties independent of sex, race, treatment type, treatment length, and status in 106 survivors with advanced melanoma [29]. In comparison, the risk of financial difficulties (*p* = 0.010) was higher in younger survivors (<65 years of age), and a higher percentage of younger survivors reported higher than expected out-of-pocket expenses due to treatment (14% vs. 2%). Only 14% of the younger and 10% of the older survivors reported satisfaction with their current financial situation. The presence of financial difficulties was moderately correlated with lower QoL.

McLouth et al. reported financial hardship in at least two domains in 52% of survivors with metastatic non-small cell lung cancer treated with CT or CT+ICIs [69]. Additionally, caregiver employment reduction was related to increased patient-reported financial hardship (68% vs. 40%) and financial distress. In addition to these studies, Lai-Kwon et al. reported EORTC QLQ-C30 financial domain results separately and observed financial difficulties in 20% of the survivors in their study conducted on 69 survivors with melanoma [44]. Johnson DB et al. reported that most long-term survivors previously treated with ipilimumab could return to work [37]. However, the exact return to work rate was unavailable in the study.

### Other outcomes addressed

Patrinely et al. included 217 survivors with melanoma, renal cell carcinoma, and non-small cell lung cancer who survived over 2 years with ICI treatment and observed increased adiposity and muscle mass during treatment, while BMI, blood glucose, and blood pressure remained stable [39] (Table 4).

**Table 4:** Financial toxicity and other outcomes

Author	Year	Country	Study type	Study design	Sample size (*n*)	Tumor type	ICI type	Inclusion criteria	Outcome	Collected data	Measurement or follow-up time	Study results
Johnson et al. [37]	2015	USA	Single center	Retrospective	33 (24 alive)	Melanoma	Ipi	Survival over 2 years after treatment	Toxicity/functional status/return to work	irAEs, physical function (ECOG)	Twenty-four months or later	ECOG 0 or 1 at 23 of 24 survivors/many patients returned to work/ongoing steroid and thyroid replacement in 33 and 38% of the survivors
Patrinely et al. [39]	2020	USA	Single center	Retrospective	217	MM, RCC, NSCLC	Pemb/Nivo/Atezo/Ave/ Ipi+Nivo	Survival over 2 years after treatment	Toxicity/HRQoL/body composition/functional status	FACT-G/IESR/NCCN-DT/body composition (CT)/irAEs	Twenty-four months or later	No dif. in BP, glucose, and BMI/increased adiposity and muscle mass/20% chronic IRAEs/younger age and need for subsequent therapy > worse QoL
Thom et al. [29]	2021	USA	Single center	Cross-sectional	106	Melanoma	Ipi+Nivo/Ipi/Pembro&Nivo/Durva	Alive at 12 months after the treatment initiation	Financial toxicity, QoL	COST, EORTC QLQ-30	The median time since ICI started was 36.4 months	Financial difficulties in 23% of the survivors/higher financial toxicity (*p* < 0.001) and financial difficulties (*p* = 0.01) in survivors <65 years of age/financial toxicity had a significant correlation with global QoL and social, emotional, and role subscales
McLouth et al. [69]	2021	USA	Single center	Cross-sectional	60	NSCLC	Pembro/Nivo/Atezo/Pembro+CT	Not specified	Supportive care needs (SCNs), financial hardship	SCNs Survey-34, the Comprehensive Score for Financial Toxicity	Median 20.8 weeks after treatment start	55% of the survivors reported unmet needs in physical, daily living, or psychological domains/52% of the patients reported financial hardship in at least two domains (material, psychological, behavioral)/40% of the patients reported cancer-related employment reduction in caregivers

Abbreviations: *APEASE*, Acceptability, Practicability, Effectiveness, Affordability, Side-effects, and Equity; *Atezo*, atezolizumab; *CMR*, complete metabolic response; *CTCAE*, *Common Terminology Criteria for Adverse Events*; *CTS*, carpal tunnel syndrome; *ECG*, electrocardiogram; *ECOG*, Eastern Cooperative Oncology Group; *EORTC QLQ-C30 PF*, European Organisation for Research and Treatment of Cancer Quality of Life Questionnaire Physical Functioning; *FACT-M*, Functional Assessment of Cancer Therapy Melanoma; *HADS*, Hospitalization Anxiety and Depression Scale; *HRQoL*, *health-related quality of life; ICI*, immune checkpoint inhibitor; *Ipi*, ipilimumab; *irAE*, immune-related adverse events; *NCCN-DT, National Comprehensive Cancer Network Distress Thermometer*; *Nivo*, nivolumab; *NSCLC*, non-small cell *lung cancer*; *Pemb, pembrolizumab; RCC*, renal cell cancer; *RCT*, *randomized controlled trial*

### Ongoing studies

Our search on the clinicaltrials.gov website retrieved a total of 14 records. Almost all studies were active and in the recruiting phase. Sample sizes were variable and spanned less than a hundred participants to studies enrolling over 4000 survivors. Nine studies specifically cited immunotherapy survivorship, while the other five studies included both survivors on treatment with ICIs or basket cohorts including survivors treated with other treatment modalities in addition to ICIs. Three studies included melanoma survivors only, and the irAEs and QoL were the most commonly evaluated survivorship domains, similar to previously published studies (Supplementary Table).

## Discussion

In this scoping review of 39 studies that included samples from real-life cohorts, we observed that QoL was impaired in survivors treated with ICIs. A significant portion of the survivors had delayed/late-onset and chronic adverse events and psychosocial problems in studies conducted in cohorts with prolonged survival with ICIs. However, most of the studies had limited sample sizes and limited follow-up, lacked an adequate control group, and did not follow the survivors longitudinally, pointing out a need for prospective studies with larger sample sizes. Additionally, several survivorship outcomes, like sexual problems, financial toxicity, and return to work, were very understudied, and further research is needed in these areas. To the best of our knowledge, our study is the first systematic review evaluating the survivorship outcomes in real-life cohorts.

In addition to the long-term burden of treatment toxicities and the effects of cancer on the body, cancer survivors are coping with psychological, physical, spiritual, and social problems [70]. Over 40% of cancer survivors have unmet survivorship needs, irrespective of treatment received [71, 72]. Furthermore, the survivors with unmet needs have a lower QoL, treatment satisfaction, and even survival [73]. Therefore, the comprehensive addressing of the survivorship issues, such as psychological problems and long-term toxicities, is paramount. However, the survivorship research with ICIs lagged, with QoL results mainly available from the clinical trials, necessitating the evaluation of survivorship in real-life cohorts. Haslam et al. estimated that around 40% of persons with cancer were eligible for treatment with ICIs according to FDA approvals as of 2019 [74]. Considering the rapid expansion of ICI indications in the last 3 years, these figures are expected to increase. Therefore, most survivors with advanced cancers and a sizeable portion of those with localized cancers treated with ICI could benefit from improvements in ICI survivorship care.

The irAEs are among the most important problems during treatment with ICIs. The frequency of all grade and severe irAEs was around 70 and 10% in the pivotal clinical trials [75–77]. The most frequent adverse events were skin and endocrine irAEs, while gastrointestinal and pulmonary irAEs were associated with higher morbidity and mortality [78]. The evidence from the real-life data confirmed these figures [79, 80]. Additionally, these observational studies demonstrated a high rate of late irAEs emerging after ICI cessation [81]. Further, these studies reported novel adverse events like carpal tunnel syndrome, uveitis, neurocognitive dysfunction, and chronic musculoskeletal problems [82–84]. However, most of these reports were case series focusing on the AE presentation and management. Further research is needed to define the prevalence of these possibly under-recognized adverse events as well as the effects of adverse events on survivors’ QoL, psychosocial problems, and functioning.

Psychosocial problems are frequent in patients with cancer, especially in survivors with advanced cancers. The available studies demonstrate a significant burden of psychosocial issues in survivors treated with ICIs [36, 66]. Even more, Rogiers et al. showed a 38% rate of anxiety and neurocognitive impairment in patients whose ICI treatments ceased after at least 12 months of disease control [35]. In addition to anxiety, depression, and sleep problems, qualitative studies evaluated the survivorship experience as a whole. They demonstrated additional complex problems like creating a new routine, issues with returning to activities of daily life, and constant feelings of uncertainty [85]. While the available studies used previously validated questionnaires like HADS to evaluate psychosocial problems [36, 60], lessons from qualitative studies point out a need to develop novel questionnaires to delineate these complex problems in survivors treated with ICIs due to the unique needs of these survivors with a lingering prognosis.

Several groups reported high rates of financial toxicity and out-of-pocket expenditures in ICI-treated survivors [29, 69]. At the same time, no study formally evaluated the return to work rates in ICI-treated survivors of working age. There is a wide discrepancy in financial toxicity across different countries due to differences across insurance policies and health economics, while all of the available studies regarding toxicity were from the USA. Therefore, reports of financial toxicity from countries with variable insurance coverage are needed. The importance of return to work is expected to increase due to the increased possibility of long-term disease control with the earlier use of ICIs in advanced cancers and the use of ICIs in the adjuvant/neoadjuvant setting and studies with large sample sizes, as well as the studies evaluating the reflections of work-related issues are required in ICI-treated patients.

Sexual problems are among the most overlooked problems in survivors treated with ICIs, with only a handful of studies with less than 40 survivors focusing only on male fertility [58]. However, considering the high rates of psychosocial problems in survivors treated with ICIs and high rates of sexual dysfunction in survivors with advanced cancers treated with chemotherapy or radiotherapy, a high rate of sexual problems could be expected for ICI-treated survivors [86, 87]. Furthermore, a recent preclinical study demonstrated decreased levels of ovarian follicles in an ICI-treated mice model [88]. The possibility of a similar female infertility problem in human studies, as well as studies evaluating the other dimensions of sexual dysfunction, is urgently needed.

The available literature highlights a need for higher quality studies in previously studied survivorship domains like QoL, toxicity, and psychosocial problems, as well as studies in understudied areas. As evident from the studies on QoL, the lack of control groups from the general population or patients treated with other treatments and the evaluation of survivorship outcomes in a single time point rather than longitudinal evaluation during the treatment were major problems in the available studies. Additionally, the unavailability of separate reporting for ICI-treated survivors in studies, including survivors treated with targeted therapy or chemotherapy in addition to ICIs, the lack of validated tools, short follow-up times in most studies, and limited sample sizes were other limitations. Prospective studies with larger sample sizes, control arms, and longitudinal follow-up are urgently needed. The longitudinal follow-up could aid in the understanding of changes in more problematic survivorship issues during the treatment course. For example, while acute irAEs could impact PROs earlier in the treatment course, work-related issues and limitations in the functional status could be more problematic in the long-term survivors.

Another critical point is the use of validated tools uniformly across studies to help extrapolate quantitative evidence and generate more reliable data in the form of meta-analyses in the future. Additionally, PRO assessments should be included in new studies evaluating ICI toxicities to delineate the effects of toxicities on the daily living of the survivors. As noted in the “Results” section of the review, the available studies did not comply with the recent irAE terminology recommendations by STIC. Later studies reporting according to these recommendations would increase the harmonization across studies and could aid in the extrapolation of the data more efficiently. Lastly, there is an urgent need for studies in understudied survivorship domains. These include but are not limited to financial toxicity, sexual problems, and work-related issues. With the expanded use of ICIs and the increase in the number of survivors in the advanced setting, more emphasis should be given to these survivorship outcomes, as evident in the review.

The main strength of this review is the comprehensive search strategy with the inclusion of several survivorship outcomes and the inclusive methodology. However, several limitations inherent to the scoping review methodology and to the included studies exist. First of all, most of the included studies had limited sample sizes and heterogeneous inclusion criteria. Albeit the broad search strategy in a scoping review methodology, the small number of identified quantitative studies limited our ability to discuss individual survivorship outcomes in more detail. The instruments to evaluate individual survivorship issues varied across the studies and this issue limited the cross-trial comparisons and quantitative synthesis of the data. Additionally, we did not filter the included studies according to study quality to be more inclusive. While this strategy was in accordance with the scoping review methodology, it limited the generalization of the results. Lastly, a significant portion of the studies evaluating the survivorship issues focused on the risk factors for irAEs instead of the effects of irAEs on PROs. Despite these limitations, the present review is the first systematic review evaluating the survivorship issues with ICIs in real-life cohorts and could serve as a guide to define the current status and delineate the areas needing further research to improve survivorship care in patients treated with ICIs.

## Conclusion

In conclusion, the available evidence demonstrates that most survivors treated with ICIs have a significant toxicity burden, lower quality of life than the general population, and a high rate of psychosocial problems. Further research is needed to delineate the survivorship issues in survivors treated with ICIs in the adjuvant setting, in tumors other than melanoma and non-small cell lung cancer, as well as efforts to gather data on the understudied survivorship issues like fertility, financial toxicity, and return to work in survivors treated with ICIs.

## **Supplementary information**

ESM 1 (DOCX 20 kb)
