# Supplementary material for: Survivorship outcomes in patients treated with immune checkpoint inhibitors: a scoping review
Source: J Cancer Surviv. 2024 Jan 4;19(3):806–45. doi: 10.1007/s11764-023-01507-w (PMC12081552; doi:10.1007/s11764-023-01507-w)
Supplement: Supplementary file 1 — (DOCX 20 kb) [file 11764_2023_1507_MOESM1_ESM.docx]

**Table.** Ongoing studies evaluating the survivorship in patients treated with immunotherapy.

| **Title** | **ClinicalTrials.gov**  **Identifier** | **Study Type** | **Status** | **Sample Size (n)** | **Target Population** | **Survivorship Issues** | **Data Collection Methods** | **Sponsor** |
| --- | --- | --- | --- | --- | --- | --- | --- | --- |
| Immune Related Toxicity and Symptom Burden in Chronic Cancer Survivors With Melanoma Receiving Adjuvant Immunotherapy With Immune Checkpoint Inhibitors | NCT04990726 | Cohort | Recruiting | 275 | Stage II-IV Melanoma | irAEs  QOL  Symptoms (Fatigue, Depression, Sleep Problems) | N/A | M.D. Anderson Cancer Center |
| An Observational Research Study for Cancer Patients on Immune Checkpoint Inhibitors, DiRECT Study | NCT05364086 | Cohort | Recruiting | 1800 | Stage I-IV Cancer (Solid or Hematologic) | iRAEs  QOL  Functional Assesment (FACT-ICM) | PROMIS  FACT-ICM  CTCAE Grading | University of Rochester NCORP Research Base |
| Bone Loss in Melanoma Survivors Receiving Immunotherapy | NCT04516122 | Cohort | Recruiting | 40 | Stage III-IV Melanoma | Bone Health | DEXA  Serum CTX and BSAP | M.D. Anderson Cancer Center |
| The Prospective Observational COMPRAYA Cohort Study (COMPRAYA) | NCT04682470 | Cohort | Recruiting | 4000 | All Cancers in Patients Between 18-39 Years at First Diagnosis | Secondary Tumors  Psychological Distress  Fertility  QOL | EORTC QLQ-C30  Second Tumors  HADS  Fertility Problems | The Netherlands Cancer Institute |
| Quality of Life and Physical Fitness After Immune Checkpoint Inhibitors | NCT03946007 | Cross-Sectional | Recruiting | 225 | Melanoma, and NSCLC patients survivimg 2 years or more after ICIs | HRQoL  Cognitive Function  Physical Fitness  Caregiver QoL  Endocrine Function | EORTC QLQ-C30  HVLT-R  COWA  TMT  6MWT  Gonadal and Pitutiary Function  CQOLC  Hand-held Dynamometer | University Medical Center Groningen |
| Symptom Burden and Unmet Supportive Care Needs in Lung Cancer Patients Undergoing First or Second Line Immunotherapy | NCT03741868 | Cohort | Completed | 60 | NSCLC (Stage IV) | QoL  Fatigue  PRO  Supportive Care Needs | NCI-PRO-CTCAE  PROMIS Fatigue  EORTC QLQ-C30  FACIT-COST  SCNSF34  PROMIST SF-Depression  Prognosis Treatment and Perceptions Questionnaire | Wake Forest University Health Sciences |
| Patient-Reported AutoImmunity Secondary to Cancer immunothErapy (PRAISE) | NCT03849131 | Observational | Recruiting | 900 | Any cancer | Severe Autoimmune Manifestations  QoL | EORTC QLQ-C30  EQ-5D-5L | University Hospital, Strasbourg, France |
| Autoimmunity After Checkpoint Blockade | NCT04119713 | Cohort | Recruiting | 300 | Any cancer | Peripheral Blood Cell Phenotype and Function  irAEs | Peripheral Blood Cell Phenotypic Analysis | University of Pennsylvania |
| Immune Checkpoint Inhibitor Toxicity Risk Prediction in Solid Tumors | NCT04871542 | Cohort | Recruiting | 2062 | Any solid tumors | irAEs  PRO  Cognitive Function  Feasibility of an ePRO App | CTCAE  PROMIS-29  PRO-CTCAE  PROMIS Cognitive Function-SF  CYTOX Score | Southwest Oncology Group |
| Cognitive Function in Patients Treated for Metastatic Melanoma With Immune Checkpoint Inhibitors | NCT04565769 | Case-Control | Recruiting | 84 | Melanoma | Cognitive Function  Fatigue  Sleep Quality  Depression  Anxiety  QoL  Sickness Behaviour | WAIS-IV  Paced Auditory Serial Addition Test  Hopkins Verbal Learning Test  Brief Visuospatial Memory Test  Trail Making Test B  Controlled Oral Word Association Test  FACIT-Fatigue  ISI  PSQI  PAOFI  HADS  SicknessQ  EORTC QLQ-C30 | Aarhus University Hospital |
| Development of an EORTC Immune Checkpoint Inhibitor-specific Quality of Life Item List: Phase 1 & 2 (EORTC ICI) | NCT05554432 | Cohort | Recruiting | 43 | Any cancer | QoL | N/A | Institut Curie |
| QUALITOP - Monitoring Multidimensional Aspects of QUAlity of Life After Cancer ImmunoTherapy, an Open Smart Digital Platform for Personalized Prevention and Patient Management | NCT05626764 | Observational | Recruiting | 3000 | Any cancer  (ICI or CAR-T) | QoL  irAEs | N/A | Hospices Civils de Lyon |
| Sleep and Immune Checkpoint Inhibitors | NCT04070651 | Cohort | Recruiting | 240 | Advanced NSCLC | Sleep Quality  Fatigue  Depressive Symptoms  QoL  Stress  Sickness Behavior | ISI  Sleep Diary  MFSI-SF  PROMIS Depression SF  EORTC QLQ-C30  EORTC QLQ-LC29  PSS  SicknessQ | Aarhus University Hospital |
| A Study of Nivolumab in Participants With Squamous Cell Carcinoma of the Head & Neck (SCCHN) - Patient Reported Outcomes (PRO) (VOLUME-PRO) | NCT05068609 | Cohort | Completed | 509 | Advanced HNSCC | PRO  QoL | EORTC QLQ-C30  EQ-5D-5L  CTSQ  WPAI:GH | Bristol-Myers Squibb |
